# Multimorbidity states associated with higher mortality rates in organ dysfunction and sepsis: a data-driven analysis in critical care

**DOI:** 10.1186/s13054-019-2486-6

**Published:** 2019-07-08

**Authors:** Zsolt Zador, Alexander Landry, Michael D. Cusimano, Nophar Geifman

**Affiliations:** 1 Division of Neurosurgery, Department of Surgery, St. Michael’s Hospital, Toronto, ON Canada; 2 Institute of Cardiovascular Sciences, Centre for Vascular and Stroke Research, University of Manchester, Manchester, UK; 3 Division of Informatics, Imaging and Data Sciences, University of Manchester, Manchester, UK

**Keywords:** Sepsis, Multimorbidity, Data analytics, Machine learning, Latent class analysis

## Abstract

**Background:**

Sepsis remains a complex medical problem and a major challenge in healthcare. Diagnostics and outcome predictions are focused on physiological parameters with less consideration given to patients’ medical background. Given the aging population, not only are diseases becoming increasingly prevalent but occur more frequently in combinations (“multimorbidity”). We hypothesized the existence of patient subgroups in critical care with distinct multimorbidity states. We further hypothesize that certain multimorbidity states associate with higher rates of organ failure, sepsis, and mortality co-occurring with these clinical problems.

**Methods:**

We analyzed 36,390 patients from the open source Medical Information Mart for Intensive Care III (MIMIC III) dataset. Morbidities were defined based on Elixhauser categories, a well-established scheme distinguishing 30 classes of chronic diseases. We used latent class analysis to identify distinct patient subgroups based on demographics, admission type, and morbidity compositions and compared the prevalence of organ dysfunction, sepsis, and inpatient mortality for each subgroup.

**Results:**

We identified six clinically distinct multimorbidity subgroups labeled based on their dominant Elixhauser disease classes. The “cardiopulmonary” and “cardiac” subgroups consisted of older patients with a high prevalence of cardiopulmonary conditions and constituted 6.1% and 26.4% of study cohort respectively. The “young” subgroup included 23.5% of the cohort composed of young and healthy patients. The “hepatic/addiction” subgroup, constituting 9.8% of the cohort, consisted of middle-aged patients (mean age of 52.25, 95% CI 51.85–52.65) with the high rates of depression (20.1%), alcohol abuse (47.75%), drug abuse (18.2%), and liver failure (67%). The “complicated diabetics” and “uncomplicated diabetics” subgroups constituted 9.4% and 24.8% of the study cohort respectively. The complicated diabetics subgroup demonstrated higher rates of end-organ complications (88.3% prevalence of renal failure). Rates of organ dysfunction and sepsis ranged 19.6–69% and 12.5–46.7% respectively in the six subgroups. Mortality co-occurring with organ dysfunction and sepsis ranges was 8.4–23.8% and 11.7–27.4% respectively. These adverse outcomes were most prevalent in the hepatic/addiction subgroup.

**Conclusion:**

We identify distinct multimorbidity states that associate with relatively higher prevalence of organ dysfunction, sepsis, and co-occurring mortality. The findings promote the incorporation of multimorbidity in healthcare models and the shift away from the current single-disease paradigm in clinical practice, training, and trial design.

**Electronic supplementary material:**

The online version of this article (10.1186/s13054-019-2486-6) contains supplementary material, which is available to authorized users.

## Background

Sepsis remains one of the most serious medical conditions with high mortality and poor prognosis. It is responsible for more than half of in-hospital deaths and is the most costly disease in healthcare constituting $20.3 billion or 5.2% of all hospitalization expenses [1]. Generally, risk assessment scores of mortality in critical care, such as the Simplified Acute Physiology Score [2] (SAPS II), Sepsis-related Organ Failure Score (SOFA) [3], or the Oxford Acute Severity Illness Score [4] (OASIS), focus on inpatient physiological data within 24 h of admission. Only SAPS II [4] and APACHE-IV [5] incorporate some pre-existing chronic conditions. Epidemiological studies, however, demonstrate substantial effect from underlying diseases, almost doubling mortality in sepsis [6–8]. Given these epidemiological findings, it is highly relevant to consider pre-existing morbidity states in assessing critical care mortality risk.

An increasingly aging population has led to a rise in chronic medical conditions. Multimorbidity, the state of suffering from more than one illness, has a prevalence between 13 and 54% depending on the surveyed population [9–11] and is associated with increased healthcare use, decreased quality of life, and higher mortality [9]. Nevertheless, the majority of current medical education fails to consider this; treatment protocols and clinical trial designs are based on the premise that patients have one disease [9, 12]. Further, the majority of drug candidates are identified in animal experiments under standardized conditions, homogenous treatment, and control groups. The subsequent clinical trials testing then aim to reproduce the same homogeneity in the recruited patients by excluding morbidity [13, 14]. We believe that the inability to account for the heterogeneity introduced by multimorbidity is at least in part why a large number of clinical trials fail. Previous work to address this challenge by identifying homogeneous clinical profiles led to changes in the management of heterogeneous conditions including sepsis [15], asthma [16], and acute respiratory distress syndrome [17].

A large volume of information is being collected in the critical care environment [18], yielding a broad range of public datasets that incorporate tens of thousands of patients [19]. This lends itself well to advanced analytics such as machine learning [20, 21], which allows detection of complex, clinically relevant patterns. Latent class analysis has become increasingly utilized in the discovery of clinically relevant patient subgroups from heterogenous datasets [15, 16, 22, 23]. This method assumes the existence of several unobserved groups within the data which share clinical properties that are mutually exclusive between groups. To address the outstanding issue of multimorbidity, we hypothesized the existence of clinically homogenous patient subgroups that share morbidity composition in critical care. We further hypothesized that distinct subgroups associate with greater risk of adverse health outcomes, such as organ failure, sepsis, and mortality related to these clinical problems. Awareness of such high-risk groups may help in anticipatory management, prognostics, and trial design. The findings may also help reshape our single-disease model in healthcare.

## Methods

### Database

We used the third edition of the Medical Information Mart in Critical Care (MIMIC3) database for our analysis. This is a single-center database containing longitudinal data on 38,597 adult patients in critical care with 53,423 distinct admissions. Further details on the database are included in (Additional file 1: Table S1). Our analysis included first-time ICU admissions for patients aged 16 or over. Readmissions to ICU were not included in the analysis.

### Definition of morbidities, organ dysfunction, and sepsis

The MIMIC3 database includes more than 15,693 distinct diagnoses, which are categorized by ICD 9 and ICD 10 codes. For a more compact representation of chronic conditions, we summarized diseases using the 30 Elixhauser categories [24] based on an algorithm provided by the authors describing the MIMIC3 database [25]. The Elixhauser categories are well established to reflect chronic diseases, and they have been validated for both ICD 9 and ICD 10 [26]. Organ dysfunction was defined based on administrative criteria [6] and integrated into the *sql* code [25] as published previously. Criteria for sepsis were defined based on those described earlier by Angus et al. [6]. Briefly, this approach defines sepsis as a combination of organ dysfunction with concomitant bacterial or fungal infection based on ICD 9 codes and has been validated prospectively using physiological and clinical features. Mortality was defined as inpatient mortality.

### Clustering and latent class analysis

We performed a preliminary analysis of similarities between diseases based on disease prevalence in the population using *k*-means clustering. We first computed disease prevalence (proportion of patients affected by the disease) in each age bracket and used Euclidean distance as similarity measure to define cluster similarities.

For the subsequent analysis of detecting subgroups of patients, we used latent class analysis (LCA) with the inputs age, admission type (elective vs non-elective), and morbidity composition (i.e., which of the 30 Elixhauser categories were present). This technique assumes the existence of unobserved (“latent”) subgroups within the study cohort and identifies them by fitting a set of mixture models to the data. In our analysis, we followed the methodological steps of determining and verifying latent subgroups as summarized by Zho et al. [27]. We chose the optimal number of subgroups based on a combination of achieving the lowest Bayesian information criteria (BIC) and Akaike information criteria (AIC) and subgroup size to be no smaller than 5% of the entire study cohort. This approach of balancing model complexity and subgroup size was adopted from previously published guidance [28], including the suggestion to remove subgroups representing significantly smaller portions of the study population. Characteristics of the latent subgroups were compared using the chi-square test for categorical variables and one-way ANOVA for continuous variables. Residual diagnostics were used to verify that the assumptions for ANOVA were not violated; expected values were calculated to verify that the assumptions for the chi-square test were not violated. We also confirmed the preferred choices between subgroups using logistic regression models assessed using the area under the receiver operator curves (AUCs) (Additional file 2: Figure S1, Table S2). Complete methodological details are described in Additional file 2. Supplementary methods.

### Network visualization

The complex relationships between morbidities were visualized using network analysis, which demonstrates associations that are otherwise difficult to appreciate. In our initial approach, when analyzing the entire critical care dataset, network nodes represented variables and the co-occurrence of variables was assessed using a relative risk (RR) measure described previously [29]. This essentially represents the risk of co-occurrence for two diseases. Associations over a significance threshold of *p* < 0.05 were included in the network. The metrics of RR is reflected by edge width, and disease prevalence is depicted by the diameter of the nodes. In the characterization of subgroups, edges were weighted by the number of patients with the disease pair normalized to the total number of patients within the subgroup. This was used in lieu of RR, as RR may underestimate co-occurrences of highly prevalent diseases as suggested by Hidalgo et al. [29].

## Results

### Heterogeneous morbidity profile in the critically ill

Cohort demographics are summarized in Additional file 1: Table S1. From a population of 36,390 patients, 83.7% of admissions were due to an emergency. There was slightly greater proportion of males (57.8%) than females. Two or more medical conditions were reported for 77.3% the patients, and nearly half of the entire study population were seniors (age 65 or over). The overall prevalence of sepsis was 37.3% (95% CI 36.7–37.9%), the organ dysfunction rate was 37.5% (95% CI 37–38%), and the overall mortality was 10.9% (95% CI 10.6–11.3%). Mortality rate recorded in patients suffering from sepsis and organ dysfunction was 21.2% (95% CI 20.3–22%) and 18.4% (95% CI 17.8–19.1%) respectively, which is comparable to previous studies in critical care cohorts [6].

**Fig. 1:**
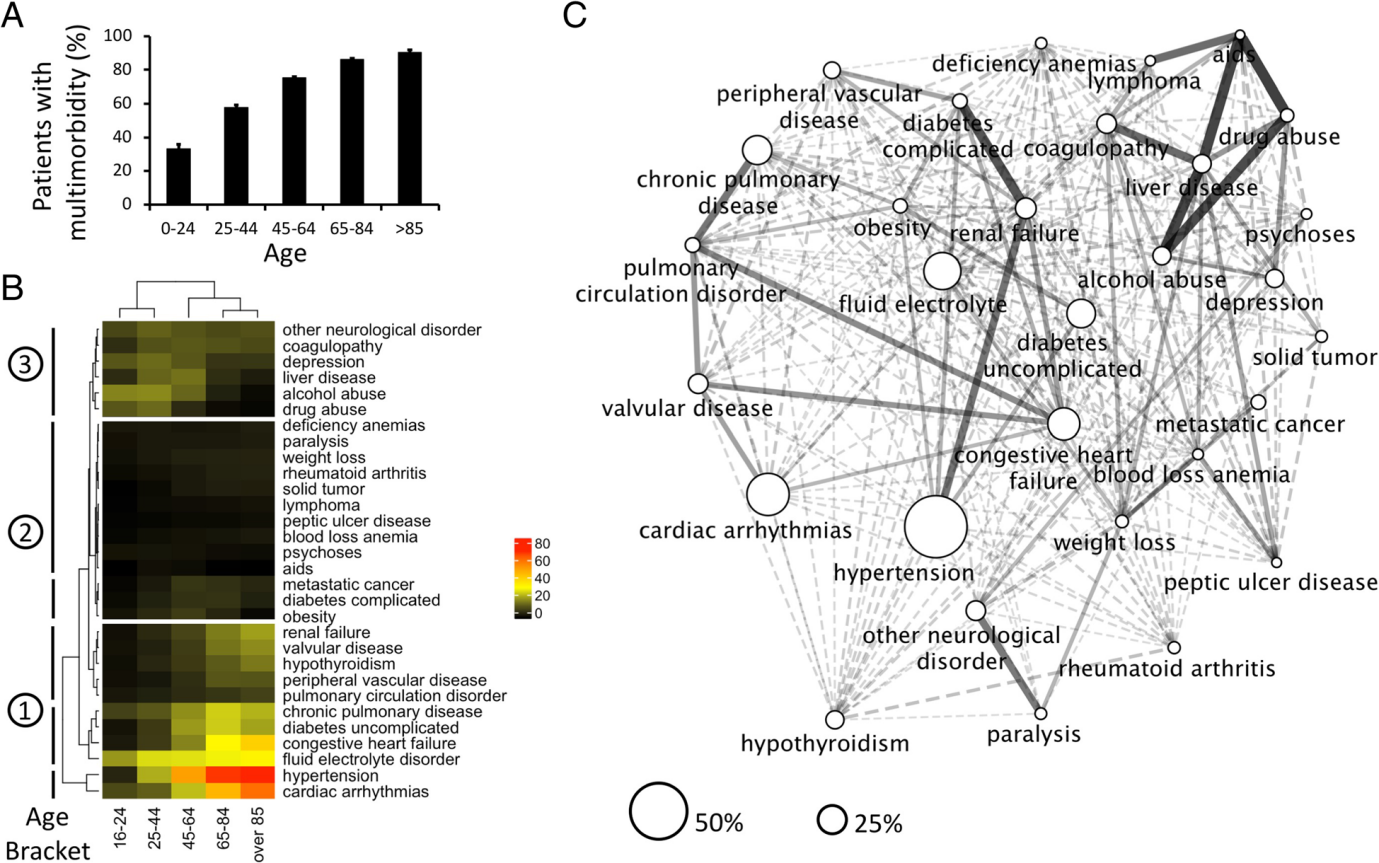
Heterogeneous morbidity profile in critical care patients. **a** Percent of patients suffering from multimorbidity progressively increases with age (average ± standard deviation). **b** Disease composition is non-homogenous over each age bracket but organizes into the following patterns: (1) diseases that are more prevalent increase progressively with age, (2) diseases with lower prevalence (0–3.8%), and (3) diseases with an earlier prevalence peak and lower prevalence with older population. Color code (right) represents percent prevalence. **c** Network discovery of disease co-occurrence shows broad range of disease associations in the critical care study cohort. Note intuitive examples such as associated cardiopulmonary conditions, diabetic nephropathy, co-occurrence of alcohol abuse, liver disease, and coagulopathy. Node size represents disease prevalence; edge width expresses relative risk for each disease pair for each disease pair; bar highlighting the multiple disease clusters in Fig 1b

The proportion of patients with multimorbidity increased steadily with age as expected [9] (Fig. 1a). However, we found that the prevalence of individual disease groups (reflected by Elixhauser categories) had distinct patterns over age brackets (Fig. 1b). Specifically, three distinct patterns were observed: one showed increasing prevalence with age, the second had diseases with lower prevalence (range 0–3.8%), and the third had peak prevalence in the 25–44 and 45–64 age bracket but lower prevalence in older age groups. Network discovery showed a variety of disease co-occurrences over the cohort suggesting frequent associations between cardiovascular with pulmonary conditions, diabetes, renal failure, and hypertension and the co-occurrence of alcohol/drug abuse, liver failure, and coagulopathy (Fig. 1c). These findings further suggested the hypothesis that distinct subgroups existed within our cohort.

### Identifying distinct multimorbidity subgroups

**Fig. 2:**
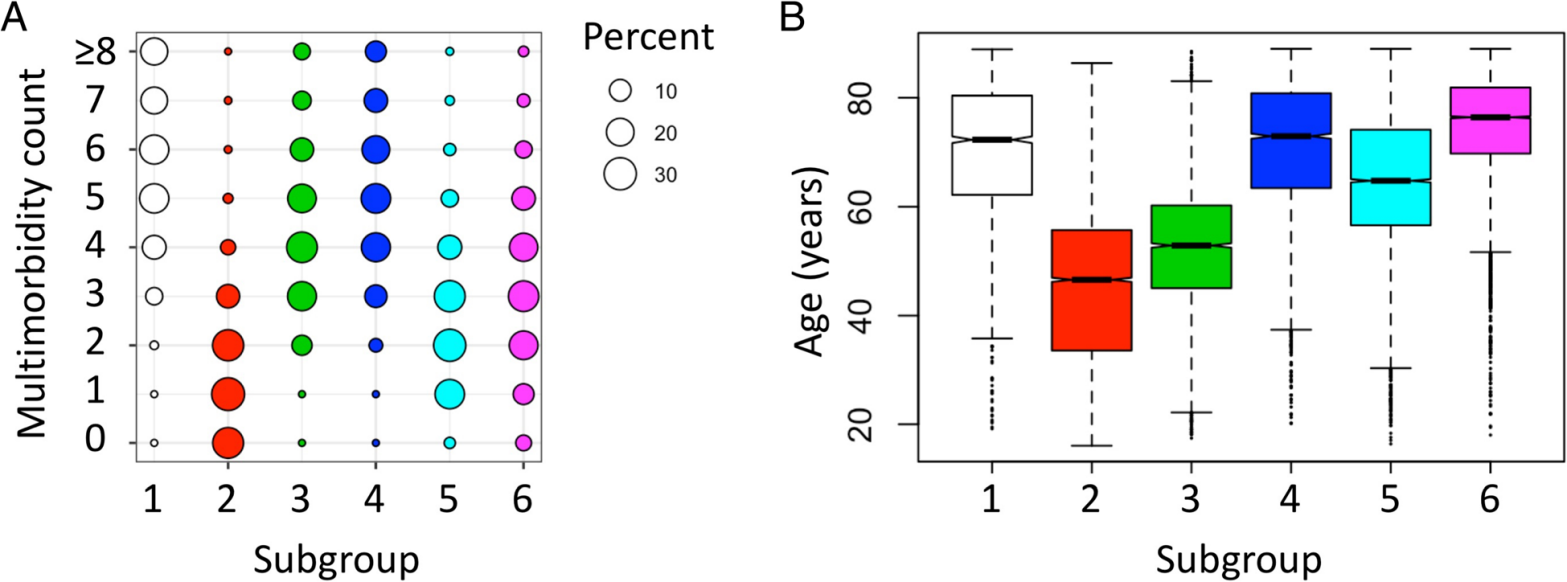
Subgroup characteristics of the six subgroups identified in critical care cohort. **a** Baloonplot summary of morbidity count for each subgroup. Highest morbidity burdens are subgroups 1, 3, 4, and 6. **b** Boxplot of age distribution in subgroups

**Fig. 3:**
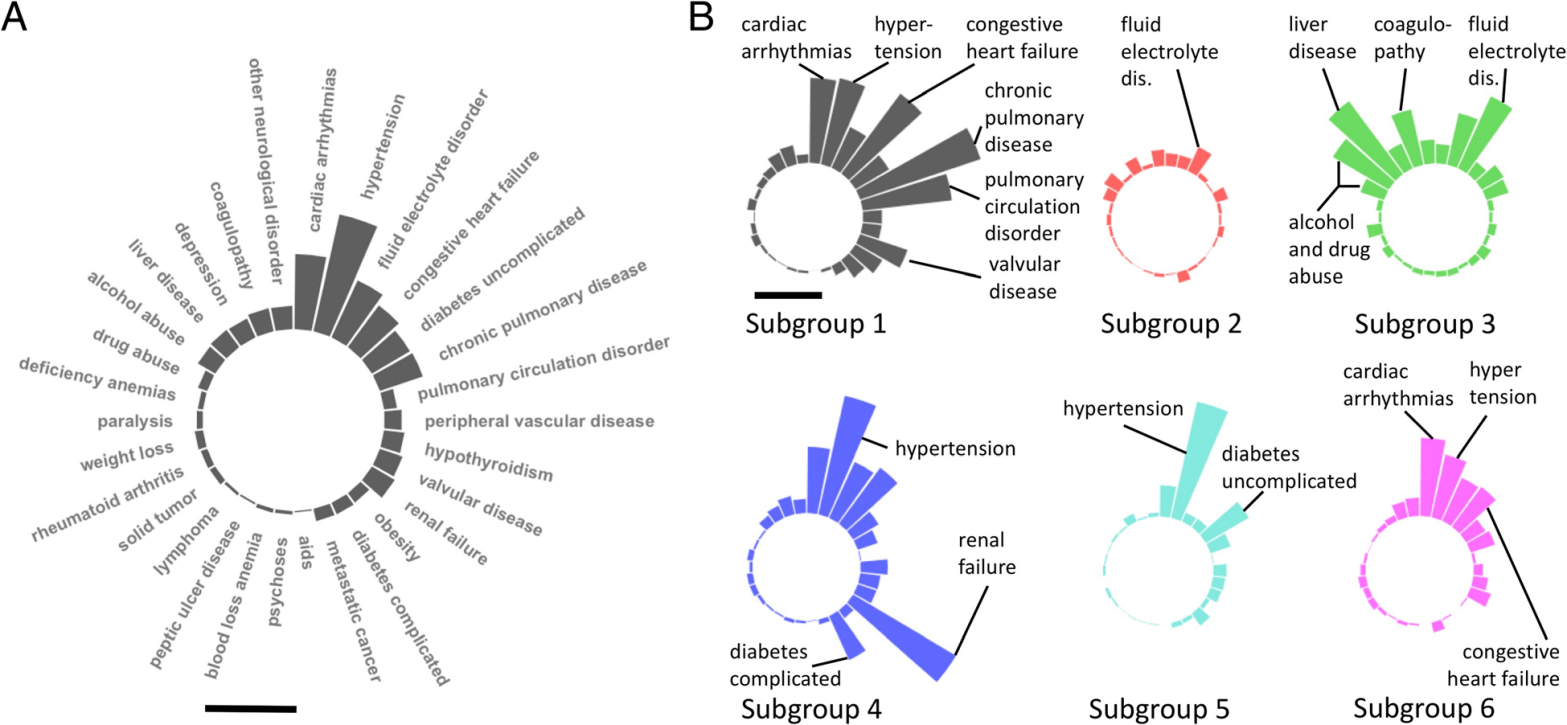
Distinct disease compositions in critical care subgroups. **a** Prevalence of Elixhauser’s morbidity categories for the entire cohort of 36,960 patients. Bar, 50% prevalence. **b** Circular barplot summary of disease composition of morbidity subgroups in critical care. Plot configuration is identical to **a**. Note the distinct patterns such as the complex cardiopulmonary profile in the cardiopulmonary subgroup (subgroup 1), health consequences of addiction in the hepatic/addiction subgroup (subgroup 3), and diabetic nephropathy with hypertension in the complicated diabetics subgroup (subgroup 4). Bar, 50% prevalence

We hypothesized that with analysis of similarities at the individual patient level, we can identify subgroups of patients with distinct demographics and disease compositions. We also hypothesized that patients belonging to certain subgroups will share vulnerability to sepsis and associated death. Using latent class analysis, we identified six subgroups of patients. The number of subgroups was defined by considering metrics of model fit and subgroup size as described in the “Methods” section. Subgroups were verified using descriptive statistics as well as simulations (see the “Methods” section and Additional file 2. Supplementary methods). Disease compositions paralleled some of the patterns suggested by the previous network discovery of the entire study cohort (Fig. 1c). Subgroup characteristics are summarized in Figs. 2 and 3 and Additional file 3: Table S3.

In the first “cardiopulmonary” subgroup, we found a high prevalence of cardiopulmonary conditions in older patients (mean age 72.3 ± 0.27) with the highest prevalence of chronic pulmonary diseases (93.86%). The second “young” subgroup had the youngest patients with the lowest point prevalence of any morbidity compared to the rest of the subgroups. The third, “hepatic/addiction” subgroup, consisted of middle-aged patients (mean age of 52.25, 95% CI 51.85–52.65) with the high rates of depression (20.1%), alcohol abuse (47.75%), drug abuse (18.2%), liver failure (67%), and coagulopathy (41.81%). This subgroup captured 70% of patients with liver disease from the entire critical care cohort of 36,960 patients. The “complicated diabetics” and “uncomplicated diabetics” subgroups both had a high prevalence of diabetes and hypertension; a large proportion of patients in the complicated diabetics subgroup suffered from renal failure (88.3%) and complicated diabetes (35.4%). The final “cardiac” subgroup was comprised of the oldest patients with a high prevalence of cardiopulmonary problems similar to the cardiopulmonary subgroup. Statistical testing with one-way ANOVA showed significant differences between all disease prevalence between subgroups (*p* < 0.001) supporting the distinct disease composition of each subgroup, the basis for identification of these subgroups.

### Multimorbidity subgroups vulnerable to sepsis and associated mortality

**Fig. 4:**
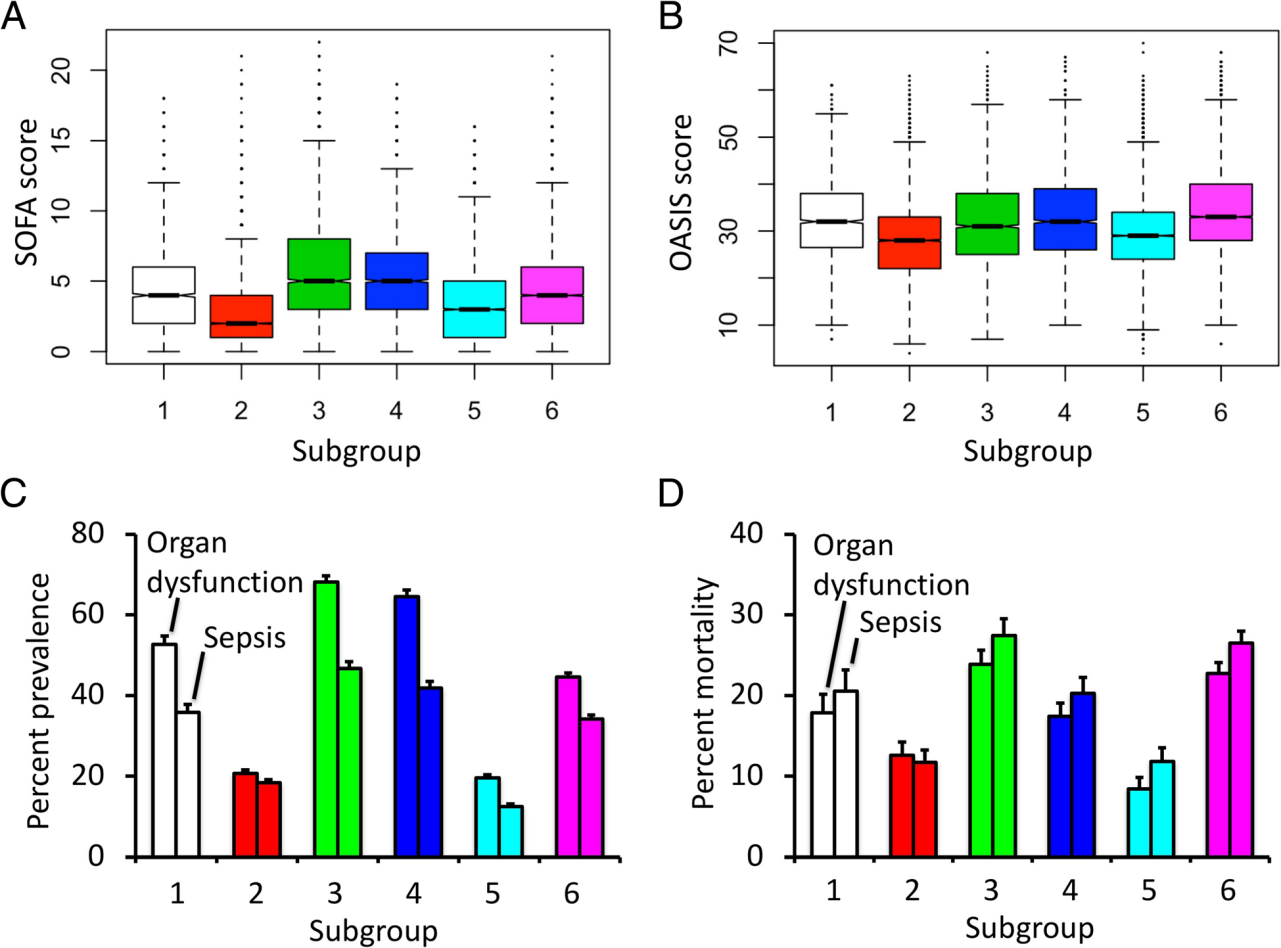
Morbidity subgroups with higher frequencies of organ dysfunction, sepsis, and associated mortality. **a** SOFA score shows most severe organ dysfunction in the hepatic/addiction subgroup closely followed by the complicated diabetics. **b** OASIS score shows similarly high mortality risk in the cardiopulmonary, hepatic/addiction, complicated diabetics, and cardiac subgroups. **c**, **d** Prevalence of organ dysfunction, sepsis, and associated mortality is highest in the hepatic/addiction subgroup (all differences are significant with multiple testing, *p* < 0.001)

We next tested if multimorbidity subgroups were different in terms of frequencies in adverse health outcomes, such as organ dysfunction and sepsis, and whether subgroups had different mortality rate associated to these clinical problems. We first established the patterns of organ dysfunctions using Sequential Organ Failure Assessment (SOFA) score which entails a system-wise observation recorded during the first 24 h of ICU stay [3]. The system-level assessment using the SOFA subscores for respiratory, cardiovascular, renal, hepatic, coagulation, and central nervous systems paralleled the morbidity profile of each subgroup (Additional file 2: Figure S2). The highest SOFA scores were detected in the hepatic/addiction subgroup closely followed by risk scores in the complicated diabetics subgroup (Fig. 4a). OASIS risk assessment score for inpatient mortality showed higher values for the cardiopulmonary, hepatic/addiction, complicated diabetics, and cardiac subgroups (Fig. 4b). The actual rate of organ dysfunction and sepsis was highest in the hepatic/addiction subgroup followed by the complicated diabetics subgroup (Fig. 4c). High mortality subgroups were also the cardiopulmonary, hepatic/addiction, complicated diabetics, and cardiac subgroups. Mortality in patients with sepsis and organ dysfunction was highest in the hepatic/addiction subgroup, almost fourfold the mortality rates in the young and uncomplicated diabetics subgroups (Fig. 4d).

### Disease co-occurrence in multimorbidity subgroups with higher mortality rates

**Fig. 5:**
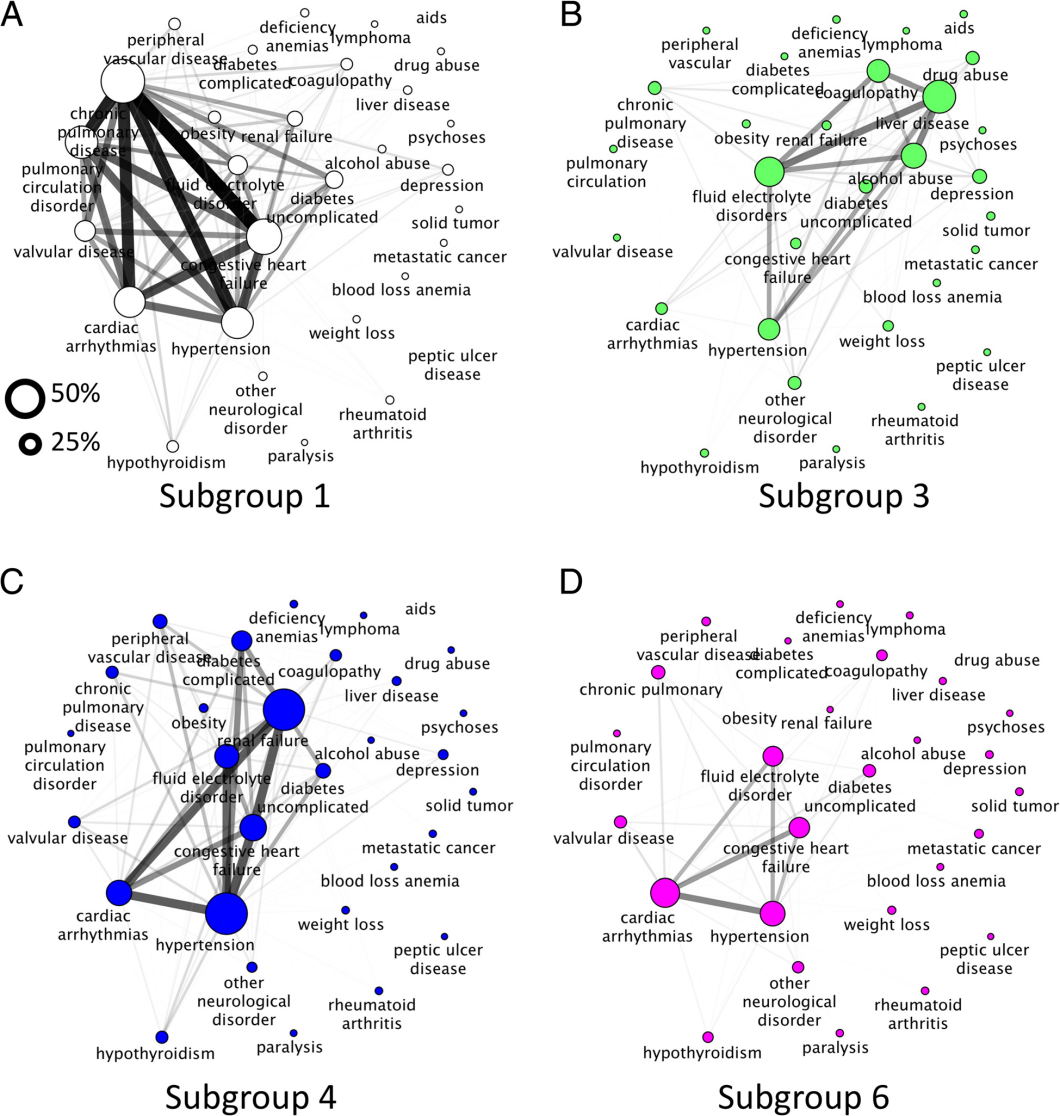
Network discovery of multimorbidity subgroups with higher rates of adverse health outcomes (organ dysfunction, sepsis, and death). Nodes represent disease prevalence, and edges express the number of patients with the disease pair normalized to the entire subgroup population. **a** Patients in the cardiopulmonary subgroup suffer from cardiopulmonary diseases as expected. **b** The hepatic/addiction subgroup has a high prevalence of health consequences of addiction. **c** For patients in the complicated diabetics subgroup, the dominant morbidity profile is diabetic nephropathy and hypertension. **d** The cardiac subgroup has a high prevalence of cardiac arrhythmias as well as other cardiac conditions

We next explored the co-occurrence of conditions for the cardiopulmonary, hepatic/addiction, complicated diabetics, and cardiac subgroups with high risk of adverse health outcomes (sepsis, organ dysfunction, and death) by visualizing prevalence and pairwise associations in a disease network (Fig. 5).

Nodes represented the Elixhauser disease categories with size defined by disease prevalence within the subgroup, and edge width was the number of patients with the disease pair normalized to the total number of patients in the subgroups (Fig. 3). Relative risk and/or Pearson’s correlation were not used in this portion of the analysis as these techniques tend to render inaccurate results for extremes of prevalence potentially resulting in under/over-estimated associations [29]. Network structure confirmed disease patterns suggested by our analysis of disease prevalence in each of the subgroups. The high mortality group consisting of younger (middle-aged) patients suggested a pattern of addiction-associated conditions (the hepatic/addiction subgroup) given the pairwise association between liver disease, coagulation disorders, alcohol excess, drug abuse, and depression. In other subgroups, the network analysis showed associations between cardiovascular-respiratory conditions (the cardiopulmonary subgroup), hypertensive-renal-diabetic with end-organ complications (the complicated diabetics subgroup), and cardiopulmonary problems (the cardiac subgroup).

Groups with lower mortality (5.31–6.02%) were either younger patients (99% below 65 years, the young subgroup) with very low disease burden or elderly with significantly higher proportion of non-emergency admissions (35.7%, the uncomplicated diabetics subgroup) than the rest of the cohort. This subgroup suffered from combination hypertensive-diabetes without end-organ complications (Additional file 2: Table S2, Figure S3).

## Discussion

Our study identified multiple clinically relevant subgroups in a critical care cohort with differing frequencies of organ dysfunction, sepsis, and associated mortality rates. In addition to the expected phenotypes of multimorbid elderly with high mortality, we also found a group of younger patients suffering from health consequences of addiction with the highest rates of sepsis and organ dysfunction. Although the association of liver failure with poor outcomes in sepsis is well established, the multimorbidity configuration it occurs in is less appreciated. These findings support the shift away from the single-disease model in healthcare to a more holistic construct, putting emphasis on considering multimorbidity composition in clinical decision-making.

As expected, we found an increasing prevalence of multimorbidity with age in line with previous population studies. However, several of our identified subgroups demonstrated distinct morbidity composition and high rates of organ dysfunction, sepsis, and associated mortality. The disease combinations within these subgroups (the cardiopulmonary, hepatic/addiction, complicated diabetics, and cardiac subgroups) were in line with previous observations and can also be interpreted along the lines of shared risk factors and patho-mechanisms. For example, chronic pulmonary disease and congestive heart failure co-occur with the highest prevalence in the cardiopulmonary subgroup; this may be explained by the concept of the cardio-pulmonary continuum [30]. The complicated diabetics subgroup is associated with high rates of diabetic nephropathy and hypertension while the cardiac subgroup represents largely elderly patients with cardiovascular diseases and a higher rate of neurological conditions.

The subgroup associated with greater rates of adverse health outcomes consisted of younger/middle-aged population who suffered from a high prevalence of alcohol and/or drug addiction, associated with liver disease and coagulopathy. While liver disease is well recognized as a risk of sepsis as well as poor outcomes, our novel findings are as follows: (1) we identify complex multimorbidity phenotypes incorporating chronic liver disease and (2) we compare it with other disease configurations in terms of sepsis prevalence and mortality. The demographics and disease composition of this high-risk group in critical care paralleled the population-level data. US Center for Disease Control and Prevention showed a peak percentage of alcohol consumption in the age bracket of 25–44 and 77% of deaths from alcoholic liver disease under the age of 65 (https://www.cdc.gov/DataStatistics/). Population studies covering 175 million hospital discharges demonstrated that the 4.5 million patients suffering from liver cirrhosis were twice as likely to die while in hospital, have sepsis as the reason for admission, and die of sepsis [31, 32]. Bacterial infections are present in up to 30% of admissions with liver failure [33]. The liver has widespread functions in responding to sepsis such as synthesis of proteins for immune, coagulation, and metabolic functions as well as scavenging of endotoxins and bacteria [34–36]. These findings parallel the higher rate of organ dysfunction traceable in our own results with the highest SOFA score in the hepatic/addiction subgroup (Fig. 4a, Additional file 2: Figure S2).

Chronic conditions have long been implicated in the disparities of sepsis prevalence [8]. The presence of comorbidities that impact immune response such as chronic renal failure, diabetes, ethanol abuse, and HIV infection was found to associate with sepsis. Furthermore, cumulative conditions were associated with greater organ failure rates. Prior studies have shown that incorporating morbidity status has improved risk assessment of mortality in patients admitted to critical care [37]. The model developed by Min et al. included a multimorbidity index, a measure that incorporates individual likelihood ratio of death for over 5000 morbidities. In their model, it is assumed that each diagnosis has an independent impact on mortality. This approach carries the advantage of representing the scale of severities within disease categories not necessarily captured by Charlson’s or Elixhauser indices (for example, not all “metastatic cancers” are lethal). Our study focused on the co-occurrence of morbidities, and thereby, our approach considers the impact diseases have on clinical outcomes in combination rather than individually. In the multimorbid population, analysis of temporal trajectories towards sepsis yielded high mortality risk profiles in patients starting off with alcohol abuse, diabetes, and cardiovascular diagnosis [1] as the initial morbidity. These results parallel our findings that multimorbidity subgroups cardiopulmonary, hepatic/addiction, complicated diabetics, and cardiac associate with high sepsis-related deaths.

Conventionally, the recruitment into clinical trials in sepsis has been based on abnormal physiological parameters implying infection as cause for critical illness. Such recruitment strategies inevitably capture a heterogenous patient group where the morbidity profile is confounded by differences in the biological response to sepsis. For example, interventions often studied by clinical trials included modifiers of the inflammatory response in sepsis such as anti-TNF, anti-IL1-Ra, anti-LPS, corticosteroids, IV immunoglobulins, and activated protein C [38]. Importantly, however, conditions such as coagulopathy, chronic liver disease [39], and diabetes [40] with end-organ complications are characterized by alterations in these target mechanisms and thus may be falsely categorized as “non-responders.” Our study represents these patients in subgroups hepatic/addiction and complicated diabetics. Such subcategorization of trial patients into clinically and biologically homogeneous subgroups can help address this confounding effect without the need for stringent exclusion criteria that render the results difficult to generalize.

It is well recognized that managing sepsis patients with liver failure is challenging and requires an individualized form of goal-directed therapy [41]. Early diagnosis and treatment are essential. The early identification of the subgroups demonstrated in our study may therefore help decision-making regarding prophylactic antibiotics, consideration of early interventions, and lower threshold for goal-directed therapy to improve outcomes in sepsis.

One potential limitation of this study is that it is based on data from a single center; therefore, composition of catchment population, departmental protocols, resources, and staffing characteristics are potential limiting to the generalizability of our results [42]. Further, it is difficult to find external validation to our results due to MIMIC3 being a unique dataset with highly detailed clinical data that is unmatched in publicly available resources. Another limitation is that we analyze a cross section of the population and therefore cannot examine causality links between subgroups and vulnerability to sepsis and organ dysfunction. For the same reason, we have no information on time of diagnosis to allow us to distinguish between pre-existing medical conditions and newly incurred diagnoses. To address this issue, diagnosis-related codes assigned for the hospitalization were incorporated to filter out diseases that are the reason for the hospital stay versus those that are not.

Part of the limitations is that ICD codes have poor reproducibility between coders which may impact the robustness of results from administrative databases [43]. However, these inaccuracies are dependent on the diagnostic category of interest, particularly how well the condition is reported and if it is covered by specific codes. For example, ICD9 codes were useful at identifying idiopathic thrombocytic purpura [44] and brain metastasis [45], both of which have specific ICD9 codes. On the other hand, ICD9 codes do not perform well for conditions without specific codes that require for example a combination of less granular code or code that is under-reported. Such examples are catheter-acquired urinary tract infection (required combination of diagnosis and procedural code) or statin-related rhabdomyolysis (lack of ICD9 code for rhabdomyolysis). The comorbidity profile in our study relies on broad disease categories of Elixhauser, which cover multiple code categories validated against ICD9 codes with good performance [24, 26]. The other labels used in our study such as organ dysfunction and sepsis were also derived based on administrative definitions. Although earlier assessments showed low coding rates (approximately 43% of acute kidney injuries for example [46]), retrospective studies from between 2005 and 2013 show improving rates of documentation in organ dysfunction [47]. There are also limitations to using the Angus criteria in our study to identify sepsis in patients. This system relies on ICD codes to establish co-occurrence of infection and organ dysfunction. Although imperfect, it has shown reasonable performance and is better compared to other administrative sepsis definitions [48, 49]. Finally, our study is limited to patients that survive long enough or are deemed appropriate for intensive care. Therefore, our mortality numbers are very likely to underestimate true figures. Nevertheless, our study uses real-world hospital data and provides a description of the population in the first 24 h in critical care, a very relevant time frame for intervention and management. This provides critical information on the risk of sepsis and associate mortality.

## Conclusions

The increasing prevalence of multimorbidity creates complexity in medical diagnostics and treatment decisions. Our study focuses on the population of patients in critical care and examines the relevance of multimorbidity in ICU outcomes including the rate of sepsis, one of the highly relevant conditions. We identify several patient groups susceptible to adverse health outcomes in critical care. The patient group with the highest rate of sepsis, organ dysfunction, and mortality was a subgroup of patients suffering from health consequences of addiction. While this work is hypothesis generating, the findings support the shift away from the current single-disease model and promote a subgroup-specific approach in medical training, treatment, and trial design.

## Additional files

Additional file 1: **Table S1.** Demographics and outcome summary of the study cohort (DOCX 22 kb)

Additional file 2: Supplementary methods. **Figure S1.** Graph summary of ROC curves depicting predictive performance of LCA input variables at differentiating a given subgroup from the remaining subgroups. **Table S2.** Table summary of classifier performance (assessed using area under the receiver operating curve, AUC) at predicting subgroup membership based on the input variables used in the LCA (age, sex, type of admission, and morbidities). **Figure S2.** Violin plot summary of organ systems impairment in the multimorbidity subgroups. **Figure S3.** Network summary of the multimorbidity subgroups with lower rates of sepsis and death. (ZIP 524 kb)

Additional file 3: **Table S3.** Summary of morbidity composition of subgroups. (DOCX 18 kb)

## Data Availability

MIMIC 3 database is available at https://mimic.physionet.org/about/mimic/. Codes used to generate data tables are accessible at https://github.com/MIT-LCP/mimic-code
