# Supplementary material for: Multimorbidity states associated with higher mortality rates in organ dysfunction and sepsis: a data-driven analysis in critical care
Source: Crit Care. 2019 Jul 8;23:247. doi: 10.1186/s13054-019-2486-6 (PMC6613271; doi:10.1186/s13054-019-2486-6)
Supplement: Supplementary file 2 — Supplementary methods. Figure S1. Graph summary of ROC curves depicting predictive performance of LCA input variables at differentiating a given subgroup from the remaining subgroups. Table S2. Table summary of classifier performance (assessed using area under the receiver operating curve, AUC) at predicting subgroup membership based on the input variables used in the LCA (age, sex, type of admission, and morbidities). Figure S2. Violin plot summary of organ systems impairment in the multimorbidity subgroups. Figure S3. Network summary of the multimorbidity subgroups with lower rates of sepsis and death. (ZIP 524 kb) [file 13054_2019_2486_MOESM2_ESM.zip › Table S2.docx]

**Supplementary table S2.** Table summary of classifier performance (assessed using area under the receiver operating curve, AUC) at predicting subgroup membership based on the input variables used in the LCA (age, sex, type of admission, and morbidities).

| Subgroup | AUC | 95% CI |
| --- | --- | --- |
| 1 | 0.9907 | 0.9899-0.9915 |
| 2 | 0.9833 | 0.9823-0.9843 |
| 3 | 0.9799 | 0.9785-0.9812 |
| 4 | 0.9909 | 0.9902-0.9917 |
| 5 | 0.9612 | 0.9592-0.9631 |
| 6 | 0.963 | 0.9611-0.9649 |
